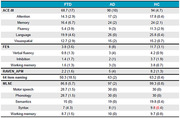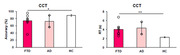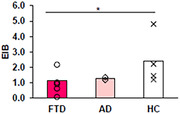# Unravelling Frontotemporal Dementia: Exploring the GABA and Glutamate Hypothesis through Semantic Deficits

**DOI:** 10.1002/alz.088279

**Published:** 2025-01-09

**Authors:** Zlatomira Georgieva Ilchovska, Akram A. Hosseini, JeYoung Jung

**Affiliations:** ^1^ University of Notingham, Nottingham UK; ^2^ Sir Peter Mansfield Imaging Centre, University of Nottingham, Nottingham UK; ^3^ Nottingham University Hospitals NHS Trust, Queens Medical Center, Nottingham UK; ^4^ Nottingham University Hospitals NHS Trust, Nottignham UK; ^5^ University of Nottingham, Nottingham UK

## Abstract

**Background:**

Semantic cognition refers to our ability to manipulate and generalize knowledge and plays a critical role in communication and daily activities. Therefore, impairments in semantic cognition can have a severe impact on quality of life (e.g., dementia). Frontotemporal dementia (FTD) is the cause of 12.5‐16.5% of all degenerative dementias. It is a heterogeneous group of clinical syndromes marked by progressive neurodegeneration of the frontal and temporal lobes – key regions for semantic cognition. Recent studies highlight the importance of addressing neurotransmitter deficits in FTD, particularly in glutamate and GABA. Though pathologically distinct, FTD syndromes share common neurotransmitter deficits – ‘GABA and glutamate hypothesis of FTD’. We investigate this hypothesis by linking GABA and glutamate changes with semantic deficits in FTD patients.

**Methods:**

We utilized MR spectroscopy, a non‐invasive, in‐vivo technique, to measure neurochemicals, including GABA and glutamate, in the brain. Our study involved 7 FTD patients, 2 typical Alzheimer’s disease (AD) patients with a symptomatic amyloid status serving as a control patient group (expected to show general cognition deficits but not specifically semantic ones, neither semantic‐specific neurotransmitter changes like those in the FTD), and 23 age‐matched healthy controls (HC). We assessed neurotransmitter concentrations in the temporal and frontal cortex, conducted computerized semantic tasks, and administered cognitive batteries assessing language, memory, attention, and executive function.

**Results:**

FTD patients showed deficits in language, memory, and executive function compared to AD patients and HCs (see Figure 1). AD patients also showed decreased general cognition compared to HC. Importantly, FTD patients performed the semantic task less effectively than ADs and HCs (see Figure 2). The balance between excitation and inhibition (EIB = glutamate/GABA) was significantly decreased in the temporal lobe in FTD patients compared to HCs (see Figure 3).

**Conclusions:**

FTD demonstrated cognitive deficits in language, memory, and executive function, along with decreased semantic task performance and reduced EIB in the temporal lobe. These preliminary findings support the GABA and glutamate hypothesis of FTD. Ongoing data collection on this CogNID project (IRAS reference: 250525, clinical trial reference: NCT03861884) will provide additional results, with more patient data to be presented at the AAIC conference.